# Safety in surgery: the role of shared decision-making

**DOI:** 10.1186/s13037-015-0068-3

**Published:** 2015-06-02

**Authors:** Alexandra E. Page

**Affiliations:** Southern California Kaiser Permanente, Musculoskeletal Health Care Solutions, California, USA

## Abstract

The only surgery without risk of complications is the one not performed. Shared decision-making (SDM) offers a process which can help a physician and patient move beyond passive informed consent to a more collaborative, patient-centered experience. By offering a balanced review of conservative and invasive treatment options, including the option of observation only, SDM provides patients an opportunity to express their personal values and goals in the context of health decisions. Thus, when the patient decides to accept the inherent risks of surgery, there has truly been an opportunity to understand and discuss all treatment alternatives.

As surgeons, we know the simplest procedure has potential not only to fail to improve the patient but potentially leave them in a position worse than when they sought your help. Informed consent stands as a buffer to inform the patients that surgery is not without risk. Regrettably, the fine print of communicating risks, benefits, and alternatives is often shorted by the enthusiasm of the patient and surgeon for the action of a surgical procedure. Considering elective procedures, referral to a surgeon may establish the expectation of surgery on both sides. For the patient, surgery may seem a clear solution to a problem and for surgeons, our training prejudices us to see the as knife as how we relieve pain and restore function. Further, the patient-doctor encounter is not structured to address patient-specific concerns about lifestyle and health care which could influence the decision of a patient to proceed with surgery. A good outcome leaves both the physician and the patient feeling better. But after a poor outcome, both parties may give more thought to what the alternatives to surgery had been. The process of Shared Decision Making (SDM) offers a mechanism to more fully incorporating patient values and perspectives to help both physician and patient feel they have come to the optimal treatment choice, whether conservative or surgical. Table 1 summarizes the apparent benefits of SDM.

**Table 1:** Benefits of shared decision-making

▪ Improved knowledge of risks and benefits by the patient
▪ Improved patient participation decision making
▪ Improved accuracy of “risk” perception
▪ Decreased decisional conflict
▪ Fewer patients remain undecided
▪ Increased patient comfort with the final decision

SDM is a communication process with the provider sharing all relevant treatment risks, benefits and alternatives, while the patient shares all preferences and values regarding his/her choices and a mutual decision on best care reflects these components. SDM is commonly employed in conjunction with the use of patient decision aids. Quality patient decision aids are designed to impart information to the patient in a manner that will allow the patient to understand their choices, comprehend the risks, benefits and alternatives to the proposed treatment and allow them in conjunction with their provider to make a decision that accords with their values and preferences.

## Informed consent vs. shared decision-making

The informed consent process involves discussion of risks, benefits and alternatives. Understanding the common risks of surgery, most surgeons develop their discussion points for the pre-op visit, may provide written material, or even digital media for education, often combining the complications as an aside to the description of the surgery and the post-op recovery. For the physician legal standards generally expect disclosure of risks, benefits and alternatives that a “reasonably prudent practitioner” would consider under like or similar circumstances. From the patient standpoint informed consent should provide information “a reasonable patient” would want to know under similar circumstances. However, in many cases rather than a true educational process, informed consent it is a signature on a sheet of meaningless small print. How often does a physician ever mention death as a possible risk of a carpal tunnel release? Yet frequently death is listed on the generic consent form for the operating room.

In contrast, SDM goes beyond passive disclosure of information to establish a process for a bidirectional exchange of information. As a tool for patient centered care, SDM has been heralded as the pinnacle of treatment [1]. Similar to informed consent, the provider shares information with the patient about the risks, benefits and alternatives of a proposed treatment. However it goes beyond to elicit values and preferences from the patient. The treatment choice can then be directed to align with these values and preferences. A meta-analysis of 115 trials of shared decision making has concluded that the shared decision making with the use of quality decision aids has led to greater knowledge of risks and benefits by the patient, more accurate risk perceptions, greater comfort with the decision, greater participation in decision making and fewer people remaining undecided [2]. SDM has the potential to become the gold standard for informed consent for many preference sensitive conditions.

One of the most important areas of SDM emerges in end-of-life discussions. Surgeons may neglect this discussion, relegating it to a medicine colleague. Part of hospital admission typically includes assessing a code status, but as with informed consent, this can be superficial discussion: would you like us to start your heart if it stops beating? Heyland et al. studied the concordance of patient and family actual preferences with the level of care orders as documented in the medical record [3]. The authors found only 30% agreement between patient end-of-life preferences based on interview with those documented in the orders. The article was followed by a compelling editorial entitled “Disregard of patients’ preferences is a medical error” [4]. Although the study was on code status, failure to accurately understand and proceed in accordance with patient wishes when operating presents an analogous, egregious medical error.

## Implementing SDM in surgical practice

SDM has the most impact in preference sensitive care. Surgical procedures such as coronary artery bypass grafting or treatment for early stage breast or for localized prostate cancer offer choices including conservative options [1,5]. Greater patient involvement in the decision making process led to greater satisfaction with the outcome for these procedures [5]. Orthopedic elective orthopedic cases such as total joint replacement have also been studied [6-11].

As a process, improving patient decision-making has been perceived as a time-consuming endeavor [12]. However, the literature suggests that the impact on appointment time may actually be minimal. A study demonstrated that orthopaedic surgeon visits scoring higher on decision-making impact took longer, but there was no significant difference between the average length of visits that did and did not meet minimal informed decision criteria [13]. The authors concluded time impact was modest, a finding supported by other studies. The 2014 Cochrane review reported that use of decision aids led to a median time increase of 2.55 min, with a range from 8 min shorter to 23 min longer [2].

The simplest SDM model involves passive presentation of a decision aid which the patient can review to generate questions. However, SDM should offer a process of questions and answers. Phone support with trained health professionals available to conduct the process has been found to be cost-effective in patient-engagement [12,14]. A study in hip and knee arthritis provided a health coach for initial consultation after receiving the decision aid, noting that the intervention patients made more efficient use of the office visit [8]. A study in the primary care setting including decision aids on back pain and orthopaedic procedures found patients receptive to mid-level providers offering the decision aid [12]. In prostate cancer, only 35% of radiation oncologists and urologists report using a decision aid as part of discussion [15]. Whether formal SDM in the office of the primary care provider, or after the referral to surgery has been done, provision of a decision aid to the patient prior to the visit can improve office efficiency. Patients opting against surgery may choose not to proceed with an orthopaedic consultation. Noting time surgeons spend addressing surgical details with patients who opt for conservative treatment, helping patients recognize their preferences earlier can allow the surgeons to focus information more effectively [6].

Infrastructure can also impact the ability for success with implementation of SDM. A study assessing SDM at multiple sites and across multiple service lines including orthopaedics found greatest success in the fully integrated system and another noted lack of a robust clinical information system as a specific barrier [12,16]. Newer demonstrations are underway to assess ability to expand into private practice, non-integrated affiliates, but lower success of the model in multispecialty pilot sites raises concerns for scalability.

## Lack of financial incentive

All of the SDM models require investment, both for decision aids and the personnel. Lack of a reimbursement mechanism for these costs creates a barrier to dissemination despite evidence of SDM as a best practice. The Center for Medicare & Medicaid Innovation under ACA provisions has facilitated growth of SDM and it is increasingly used as a standard for different accrediting bodies [17,18]. Commercial payers, enticed by the potential for cost savings as well as patient satisfaction, have the ability to structure insurance products to stimulate use of SDM, but the physician incentive remains unproven [18-20].

## SDM as a tool to lower liability risk

Most surgeons have learned the maxim “*Never talk a patient into surgery*”. A patient reluctant to undergo surgery who feels “talked into” the procedure is far more likely to be unhappy with a poor outcome. Informed consent has been the classic documentation of physician-patient communication on risks, benefits, and alternatives for treatment and surgery. The enhancements of SDM offers improved patient comprehension of risks and benefits and with this, frustration with adverse outcomes can be decreased. Eliciting the personal preferences of the patient then working to align these values and preferences with treatment decision strengthens the therapeutic alliance and is more protective of the provider in a medico legal context [21]. Poor communication by the provider and inadequate knowledge on the part of patient are often precursors for medical liability claims [22].

The impact of a formal SDM process in ligation has been studied. Using a mock jury for a simulated malpractice case involving screening decisions for prostate cancer, documentation of discussion was not felt to meet the standard of care by 28% of the focus group. However, documentation of the use of a decision aid reduced this to 6%, with the authors concluding that use of a decision aid offered the highest medical-legal protection [23].

Legislation is currently moving toward SDM as a model for patient-centered care. For example, Washington State has promoted it as a mechanism to achieve better informed consent. In 2007, this state passed legislation supporting SDM, noting it as evidence of informed consent. In 2009, California, Connecticut, Maine, Minnesota, and Vermont had legislative action on SDM bills [19]. At this time there are no known liability insurance premium reductions for use of SDM, but this has been discussed as a possible incentive. ACO regulations, PCMH, and value based insurance design commonly call for the use of SDM to more fully inform and engage the patient.

## Take-home message

Patient safety includes the entire spectrum of the care cycle, starting with the decision to undergo surgery. It is easy to agree that performing an unnecessary surgery violates patient safety. For preference-sensitive surgeries, determining the true indication of the surgery should be based on mutual discussion of risks, benefits, and alternatives combined with the patient’s preferences and values. This shared decision making process ensures that the patient undertakes the risks of surgery with understanding of options in the context of their own life. While it can’t eliminate the risks of surgery, SDM can make both surgeon and patient more comfortable with proceeding and with facing any complication that could arise.
